# Data on the effect of current density relationship on the super-alloy composite coating by electrolytic route

**DOI:** 10.1016/j.dib.2018.03.084

**Published:** 2018-03-26

**Authors:** O.S.I. Fayomi, A.A. Daniyan, L.E. Umoru, A.P.I. Popoola

**Affiliations:** a Department of Mechanical Engineering, Covenant University, P.M.B. 1023, Ota, Nigeria; b Department of Materials Science and Engineering, Obafemi Awolowo University, Ile-Ife, Nigeria; c Department of Chemical, Metallurgical and Materials Engineering, Tshwane University of Technology, P.M.B. X680, Pretoria, South Africa

**Keywords:** Modified, Electrocodeposition, Nanocomposite, Current, Coating

## Abstract

In this work, a detail effect of nanoparticle loading and improved process parameter on the synthesis of modified Zn-TiO_2_ electrocodeposited nanocomposite coating was presented. The coatings were performed at constant time of 20 minute at a stirring rate of 400 rpm at temperature of 70 °C. The effect of particle loading and input current on the properties of the electrocodeposited Nanocomposite was studied. The co-deposition was carried out at a current interval between 1.0 and 1.5 A for the coating period. The basis of bath formulation as it quantitatively and qualitatively affects the coating system was put into consideration. Hence, the electrocodeposition data for the coating properties and coating per unit area were attained. Also,the effect of annealing heat treatment on the hardness properties of the nanocomposite coatings was carried out.The annealing temperature used was 250 °C so as to ascertain the thermal stability of the coatings and to achieve homogenisation of the coating system. The weight gained under difference coating condition were attained and could be applied using modified Zn-TiO_2_ electrocodeposited nanocomposite coatings as an effective and safe alternative coating to chromium and other harmful coatings.

**Table:** 

Subject area	*Materials Science Engineering*
More specific subject area	*Surface Engineering*
Type of data	*Table, image*
How data was acquired	The coating took place in a fabricated electrocodeposition sequence cell containing stepsin accordance to the principle of electrolytic co-deposition path from pre-treatment to post treatment. The coating thickness, weight gained, coating per unit area were measured using coating thickness gauge and weighing balance for the weight gain. The coating per unit area was obtained from the calculated value of the coating thickness for each value of co-deposited matrix.
Data format	Raw, Analyzed
Experimental factors	The particles were measured properly and the pH of the electrolyte was measured before the deposition was done and required data acquired.
Experimental features	The depositions were performed at constant time of 20 min at a stirring rate of 400 rpm at the suitable temperature of 70 °C. The effect of particle loading and current density on the properties of the electrocodeposited Nanocomposite. The basis of bath formulation as it quantitative and qualitative affect the coating system was put into consideration.
Data source location	Department of Chemical, Metallurgical and Materials Engineering, Tshwane University of Technology, Pretoria, South Africa and Department of Materials Science and Engineering, Obafemi Awolowo University, Ile-Ife, Nigeria
Data accessibility	Data are available within this article

## Value of the data

• The given data will show authors in the field of surface Engineering the relationship and effect between the ceramic nanoparticles and metallic electrolyte and the constant metallic matrix nanocomposite bath in a given engineering application.
• The data obtained for the zinc matrix electrolyte as well as the incorporated nanoparticulates can be used as inference to determine the anomalous metal matrix co-deposition coating for other intended nanocomposite coating.
• The data can be used to examine the relationship between the process variable for instance (current and particle loading) as it influences the nature of coating characteristic produced.
• The data could be used at investigating the coating progress between the coating thickness, weight gain and the surface area of the incorporated nanoparticulates.
• The data acquired can be used in investigating the trend in microhardness profile of the Matrices of Nanocomposite either at room or elevated temperature for surface application.

## 1 Data

The coating thickness, weight gained, coating per unit area at constant deposition time were collected and a distinctive set of experimental frame work data were produced. The depositions current was carried out at varying values of 1.0–1.5 A at a stirring rate of 400 rpm at 70 °C temperature. The data acquired from elemental analysis of the mild steel is presented in Table 1. The coating depositions was run with two opposing zinc anode to enhance uniform coating. Also, data showing coating variable in term of current and loading of particles was gathered (See Tables 2 and 3).

**Table 1:** Data showing chemical composition of as-received mild steel.

**Element**	**% Content**	**Element**	**% Content**	**Element**	**% Content**
C	0.134	Mo	0.083	Ti	< 0.002
Si	0.119	Ni	0.019	V	0.0048
Mn	0.237	Cu	0.044	W	0.024
P	< 0.003	Al	0.050	B	> 0.016
S	> 0.156	Co	0.012	Sn	0.0046
Cr	0.094	Nb	< 0.005	Fe	97.70

**Table 2:** Electrocodeposition parameters and results for the coated mild steel.

**Sample**	**Time (min)**	**Coating thickness (μm)**	**Weight gained (g)**	**Current (A)**	**Coating per unit area (g/cm**^**2**^**)**
Zn-1.0A	20	45	0.34	1.0	0.024
Zn-1.5A	20	115	0.57	1.5	0.031
Zn-TiO_2_-1.0A	20	25	0.42	1.0	0.026
Zn-TiO_2_-1.5A	20	20	0.53	1.5	0.028
Zn-TiO_2_-WO_3_-8*g*-1.0A	20	25	0.45	1.0	0.024
Zn-TiO_2_-WO_3_-8*g*-1.5A	20	60	0.48	1.5	0.030
Zn-TiO_2_-WO_3_-15*g*-1.0A	20	35	0.41	1.0	0.023
Zn-TiO_2_-WO_3_-15*g*-1.5A	20	40	0.51	1.5	0.036
Zn-TiO_2_-ZnO-8*g*-1.0A	20	40	0.39	1.0	0.022
Zn-TiO_2_-ZnO-8*g*-1.5A	20	45	0.62	1.5	0.035
Zn-TiO_2_-ZnO-15*g*-1.0A	20	25	0.38	1.0	0.025
Zn-TiO_2_-ZnO-15*g*-1.5A	20	125	0.60	1.5	0.031
Zn-TiO_2_-SnO_2_-8*g*-1.0A	20	60	0.43	1.0	0.022
Zn-TiO_2_-SnO_2_-8*g*-1.5A	20	40	0.54	1.5	0.038
Zn-TiO_2_-SnO_2_-15*g*-1.0A	20	45	0.38	1.0	0.022
Zn-TiO_2_-SnO_2_-15*g*-1.5A	20	95	0.66	1.5	0.037
Zn-TiO_2_-WO_3_-ZnO-SnO_2_-8*g*-1.0A	20	15	0.37	1.0	0.024
Zn-TiO_2_-WO_3_-ZnO-SnO_2_-8*g*-1.5A	20	20	0.64	1.5	0.041
Zn-TiO_2_-WO_3_-ZnO-SnO_2_-15*g*-1.0A	20	35	0.42	1.0	0.020

**Table 3:** The values of the coatings’ hardness before and after annealing thermal treatment at 250 °C for 5 h.

**Sample no.**	**Sample**	**Unheated**	**Heated**
1	Zn-1.0A	114	143
2	Zn-1.5A	121	154
3	Zn-TiO_2_-1.0A	129	145
4	Zn-TiO_2_-1.5A	130.5	155.5
5	Zn-TiO_2_-WO_3_-8*g*-1.0A	139	145.5
6	Zn-TiO_2_-WO_3_-8*g*-1.5A	188.5	145
7	Zn-TiO_2_-WO_3_-15*g*-1.0A	237.5	250
8	Zn-TiO_2_-WO_3_-15*g*-1.5A	153	137
9	Zn-TiO_2_-ZnO-8*g*-1.0A	121.5	175.5
10	Zn-TiO_2_-ZnO-8*g*-1.5A	118	169.5
11	Zn-TiO_2_-ZnO-15*g*-1.0A	136.5	163
12	Zn-TiO_2_-ZnO-15*g*-1.5A	127.5	173
13	Zn-TiO_2_-SnO_2_-8*g*-1.0A	140	170.5
14	Zn-TiO_2_-SnO_2_-8*g*-1.5A	126	210.5
15	Zn-TiO_2_-SnO_2_-15*g*-1.0A	98.95	187
16	Zn-TiO_2_-SnO_2_-15*g*-1.5A	148	171
17	Zn-TiO_2_-WO_3_-ZnO-SnO_2_-8*g*-1.0A	165	159
18	Zn-TiO_2_-WO_3_-ZnO-SnO_2_-8*g*-1.5A	129	145.5
19	Zn-TiO_2_-WO_3_-ZnO-SnO_2_-15*g*-1.0A	125.5	174.5
20	Zn-TiO_2_-WO_3_-ZnO-SnO_2_-15*g*-1.5A	122.5	186.5

## 2 Experimental design, materials and methods

**Fig. 1:**
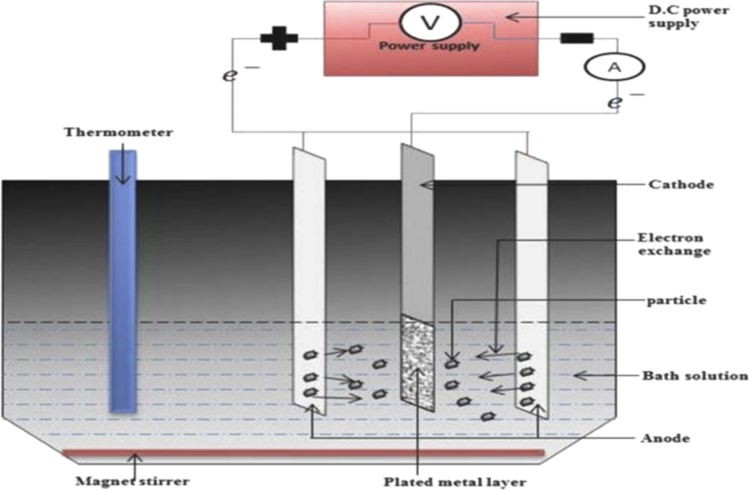
Schematic diagram of electrocodeposition system.

**Fig. 2:**
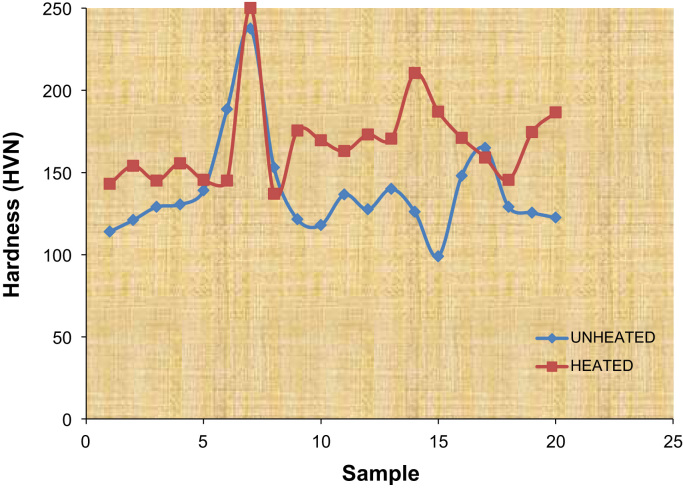
Hardness plots for all the coatings before and after homogenising annealing at 250 °C for 5 h.

An electrocodeposition system used for this set up is shown in Fig. 1. The dimension of the mild steel (substrate) used was 45 mm × 40 mm × 20 mm. Zinc sheets of 85 mm × 45 mm × 5 mm with 99.99% were prepared as anodes as described [1]. The mild steel specimens were polished mechanically, degreased and rinsed with water as described [2], [3]. Powder purchased from Sigma Aldrich was used as received. The bath formulations were prepared a day before and stir continuously at the rate of 400 rpm to obtain homogeneous mixture. The bath compositions used for the different coating matrix is as follows: ZnCl_2_ 120 g/l; KCl 30 g/l; TiO_2_ nanoparticles 20 g/l; WO_3_, ZnO, and SnO_2_nanoparticles 8–15 g/l each; 2- Butyne 1,4 diol 0.5 g/l; Cetylpridinium Chloride 0.5 g/l and 10 g/l of Thiourea. KCl was added to increase the conductivity of the electrolyte, 2- Butyne 1,4 diol and Cetylpridinium Chloride were added as surfactants to reduce the surface tension of the solution which in turn lower the surface energy, so as to give good adhesion. The dispersion reinforcement behaviour which often causes change in coating performance [4], [5], [6] helps to obtained coating thickness, weight gained, coating per unit area generated and presented in Fig. 2.
