# Supplementary material for: Data on the effect of current density relationship on the super-alloy composite coating by electrolytic route
Source: Data Brief. 2018 Mar 26;18:776–80. doi: 10.1016/j.dib.2018.03.084 (PMC5996728; doi:10.1016/j.dib.2018.03.084)
Supplement: Supplementary file 1 — Supplementary material [file mmc1.docx]

***COVER LETTER***

*24th November, 2017*

*The Editor-in-Chief*

*Data in Brief*

*Dear Sir,*

*This serve to notify you that the manuscript is original of the authors work and there is no conflict of interest of any kind regarding the manuscript Data on the effect of current density relationship on the super-alloy composite coating by electrolytic route*

*Sincerely yours,*

*Dr. OSI Fayomi*

*Department of Mechanical Engineering*

*Covenant University
Ota,*

*Nigeria*
